# Grafting of Crown Ether and Cryptand Macrocycles on Large Pore Stellate Mesoporous Silica for Sodium Cation Extraction

**DOI:** 10.3390/molecules28124622

**Published:** 2023-06-07

**Authors:** Paula Duenas-Ramirez, Caroline Bertagnolli, Robin Weiss, Joëlle Bizeau, Loïc Jierry, Philippe Choquet, Ariane Zaloszyc, Sylvie Bégin-Colin, Damien Mertz

**Affiliations:** 1 Institut de Physique et Chimie des Matériaux de Strasbourg (IPCMS), UMR-7504 CNRS-Université de Strasbourg, 23 Rue du Loess, 67034 Strasbourg, France; pduenasramirez@group-gac.com (P.D.-R.); joelle.bizeau@ipcms.unistra.fr (J.B.);; 2 Institut Pluridisciplinaire Hubert Curien (IPHC), UMR 7178 CNRS-Université de Strasbourg, 25 Rue Becquerel, 67087 Strasbourg, France; caroline.bertagnolli@unistra.fr; 3 Institut Charles Sadron (ICS) CNRS UPR 22, 23 Rue du Loess, 67034 Strasbourg, France; robin.weiss@unistra.fr (R.W.);; 4 UF6237 Imagerie Préclinique, Pôle d’Imagerie, Hôpitaux Universitaires de Strasbourg, 1 Avenue Molière, 67098 Strasbourg, France; pchoquet@unistra.fr (P.C.); arianezaloszyc@gmail.com (A.Z.); 5 Institut de Chimie et Procédés pour l’Energie, l’Environnement et la Santé (ICPEES), UMR-7515 CNRS-Université de Strasbourg, 25 Rue Becquerel, 67087 Strasbourg, France

**Keywords:** large pore mesoporous silica, ether crown, cryptand, sodium extraction, chelating nanoparticles

## Abstract

Regulation of the sodium cations level in the case of renal failure diseases is a very challenging task for clinicians, and new pollutant extractors based on nanomaterials are emerging as potential treatments. In this work, we report different strategies for the chemical functionalization of biocompatible large pore mesoporous silica, denoted stellate mesoporous silica (STMS), with chelating ligands able to selectively capture sodium. We address efficient methods to covalently graft highly chelating macrocycles onto STMS NPs such as crown ethers (CE) and cryptands (C221) through complementary carbodiimidation reactions. Regarding sodium capture in water, C221 cryptand-grafted STMS showed better capture efficiency than CE-STMS due to higher sodium atom chelation in the cryptand cage (Na^+^ coverage of 15.5% vs. 3.7%). The sodium selectivity was hence tested with C221 cryptand-grafted STMS in a multi-element aqueous solution (metallic cations with the same concentration) and in a solution mimicking peritoneal dialysis solution. Results obtained indicate that C221 cryptand-grafted STMS are relevant nanomaterials to extract sodium cations in such media and allow us to regulate their levels.

## 1. Introduction

Sodium is the principal extracellular cation in the human body and fluids, and its concentration is regulated by the hypothalamic–pituitary axis (neuroendocrine system) and the kidneys. Hypernatremia (serum [Na^+^] > 145 mM) can be caused by renal or non-renal loss of water or by concentrated salt intake. Hypernatremia leads to hyperpnea, muscle weakness, insomnia, lethargy and, ultimately, coma [1,2]. In order to manage hypernatremia, one has to first identify the cause [3] and then administrate a replacement solution. More anecdotally, continuous replacement therapy has been reported in severe hypernatremia. In the case of chronic kidney disease, patients present with sodium and volume overloads, which can eventually be associated with dysnatremia [4] and lead to hypertension and contribute to impaired cardiovascular outcomes [5,6]. Sodium removal by chronic peritoneal dialysis or hemodialysis is challenging, and a salt-restricted diet is frequently recommended in patients that require dialysis. In order to improve sodium balance, varying dialysis time and decreasing sodium content have been explored, but can lead to adverse effects such as hypotension [7,8,9]. Moreover, a proportion of the sodium is stocked on the skin and is hardly removable [10]. On the other hand, few investigations have been developed on sodium removal as its concentration has to be strongly mastered as hyponatremia is critical for the patient. Thus, it has to be ensured that the homeostatic levels of this cation remain superior to a limited value for the safety of such treatment because depletion of some relevant ions could be risky, and depletion of sodium could produce cardiovascular problems such as hypotension episodes [11]. Solutions for sodium purification in industry and laboratories exist but present many disadvantages such as toxic side products, nonspecific uptake, and are often expensive. Some examples of organic extractors for sodium are charged membranes [12] and ionic liquids (e.g., monensin) [13]. Unfortunately, they are not compatible with medical treatments. Therefore, there is currently a need to develop new solutions for controlled sodium uptake.

Among removal processes ensuring a controlled uptake of cations, those involving highly chelating molecules such as crown ethers and cryptands (Nobel Prize 1984: Lehn, Pedersen, and Cram) have been demonstrated as highly selective for different ions. In 1967, Pedersen accidentally synthesized and isolated the first crown ether: the dibenzo 18-crown-6, able to selectively capture potassium cations [14]. The increase in the number of known crown ethers helped to understand and appreciate the limits of size and the study of binding properties of these new macrocycles. Based on the discovery of Pedersen, Lehn and his team developed cryptands. Crown ethers are cyclic compounds consisting of a ring of several ether groups; meanwhile, cryptands contain two bridgehead atoms (nitrogen, phosphorous, etc.) connected by three bridges [15]. The advantage of crown ethers over cryptands is the easiness of the synthesis process, so they are less expensive. On the other hand, cryptands are more selective for certain ions. The thermodynamical stability of those complexes depends on the match of the cavity and cation size. Generally, cryptands have a bigger stability constant because the rigidity of the cage matches better with the ionic radii. Despite such molecules appearing as ideals systems to chelate and extract any cation from a biological media, several issues, that are quite similar to those existing for the administration of molecular drugs from drug delivery nanocarriers, can also be addressed for such depollution. Indeed, given their small molecular size, their use in complement to hemodialysis or peritoneal dialysis would be rather limited because of their rapid renal filtration or easy crossing of peritoneal membranes (given the pore size channels in the peritoneal membrane that are between 2 and 4 Å for the ultra-small pores, 40–55 Å for the small pores and 150–300 Å for the big pores) [16,17,18]. Therefore, anchoring such chelating ligands on highly mesoporous nanomaterials would solve the various issues addressed above.

Furthermore, there are very few studies reporting nanomaterials for efficient and selective sodium removal, and they are aimed to desalination processes. For instance, a study on the magnetically induced extraction of metallic cations such as sodium, calcium, and potassium from seawater was reported with a moderate optimum ion removal ratio of ca. 7% by employing magnetite nanoparticles combined with clinoptilolite, a common zeolite [19]. In another work, the selective removal of Na^+^ was also achieved using NaTi_2_(PO4)_3_ nanoparticles embedded into a carbon nanotube hollow fiber for the selective removal of Na^+^ during the capacitive deionization of salty water [20]. These materials have an ion adsorption capacity of ca. 1–2 mmol·g^−1^ per adsorption and desorption cycle, repeated several times. However, such a composite requires immobilisation in the electrodes under constant applied voltage and would not be adapted for biomedical applications. Furthermore, to the best of our knowledge, there are no examples reported in the literature making use of mesoporous silica bearing highly chelating molecules for the selective extraction of sodium in aqueous solution. The functionalization of silica materials has already been assessed for metallic pollutant capture, especially regarding the removal of heavy metals from water. For instance, thiols functionalized on a silica surface were used to extract heavy metals such as mercury, cadmium or lead [21,22]. The functionalization of silica with amine groups also allowed the capture of hazardous pollutants. Hence, polyethyleneimine (PEI)-coated mesoporous silica was tested to extract cadmium and nickel [23], while aminopropyl groups were grafted onto various mesoporous silica (MS) surfaces to remove the chrome, arsenic, mercury [24], or nickel, cadmium and lead [25]. It is worth noting that, in these works, the chosen groups were not specific, and functionalization with highly chelating and selective ligands is still a challenge. 

**Scheme 1:**
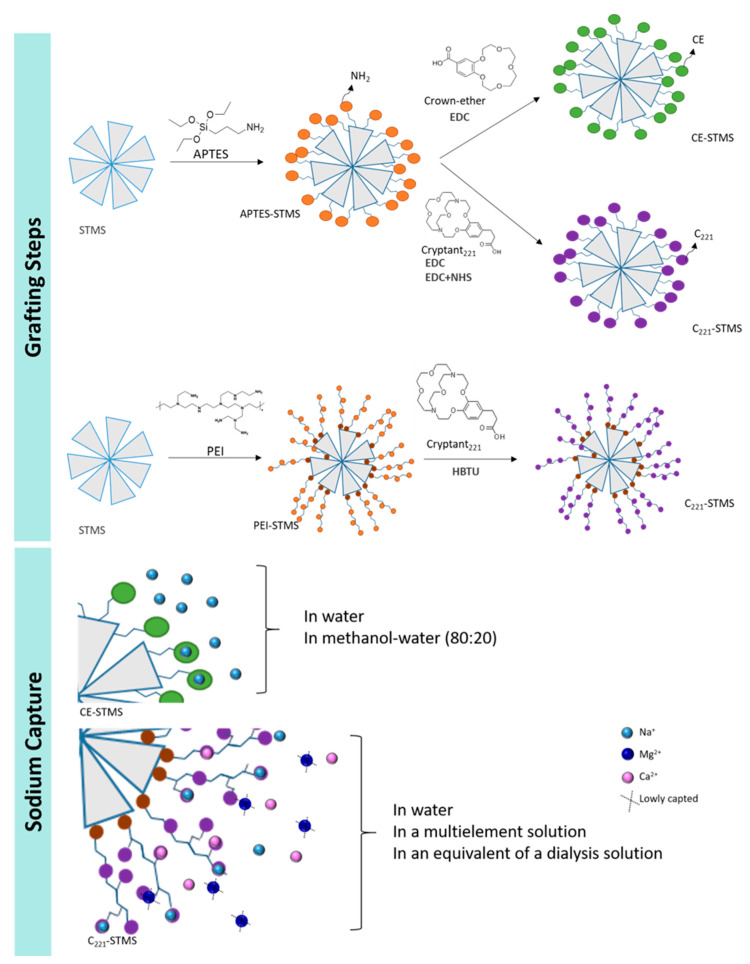
General scheme of the grafting steps of the highly chelating macrocycles CE and C_221_ and the subsequent conditions used to investigate sodium removal.

In that context, we developed new functionalized and original submicron-sized materials based on the covalent grafting of high specific sodium chelators on large pore stellate mesoporous silica (STMS) NPs [26,27]. Such stellate NPs were previously chemically modified with a variety of functionalities for iron extraction [28], protein coating/release applications [29,30], or in vivo bioimaging [31] or when embedded with an iron oxide core for magnetic or photonic hyperthermia [32,33]. Here, such stellate silica NPs functionalized with crown ethers or cryptands could efficiently contribute to removing sodium from biological environments. Hence, we first achieved the grafting of carboxylated 15-crown-5 ether (CE) on aminosilane grafted-STMS via an EDC carbodiimidation reaction. Then, in a second approach, the covalent grafting of carboxylated cryptand [2.2.1] (named cryptand_221_ and abbreviated C_221_) was evaluated on both aminosilane and polyethyleneimine(PEI)-coated STMS by different carbodiimidation reactions. Both macrocycles are well-known for their capacity to strongly bind sodium cations (CE: cavity radius 1.7–2.2 Å [34] /C_221_: 1.1 Å, ionic radius of sodium 0.98 Å [35]). Thus, in this work, we present different chemical strategies to efficiently graft CE and C_221_ at the STMS surface, and these CE-STMS and C_221_-STMS are then investigated for Na^+^ uptake properties in NaCl solutions under different conditions. Regarding CE-STMS, sodium cations capture is first tested in co-solvent methanol–water (80/20% *v*/*v*) and then in water by varying Na:CE stoichiometry and pH. Regarding C_221_-STMS, they are assessed only in water media (0.9 and 4.7 Na eq). Furthermore, as the dialysis solution in peritoneal dialysis is generally a multi-ionic aqueous solution (Ca^2+^, Na^+^, Mg^2+^ and Cl^−^), sodium selectivity is tested in a multi-element aqueous solution (at the same concentration) and in equivalent concentrations to a dialysis solution. The next scheme summarizes the different synthesis strategies to graft the ligands and the performed experiments on the systems (Scheme 1).

## 2. Results and Discussion

### 2.1. Crown Ether Functionalization on STMS and Sodium Extraction

#### 2.1.1. Grafting of Crown Ether on STMS Nanoparticles

**Figure 1:**
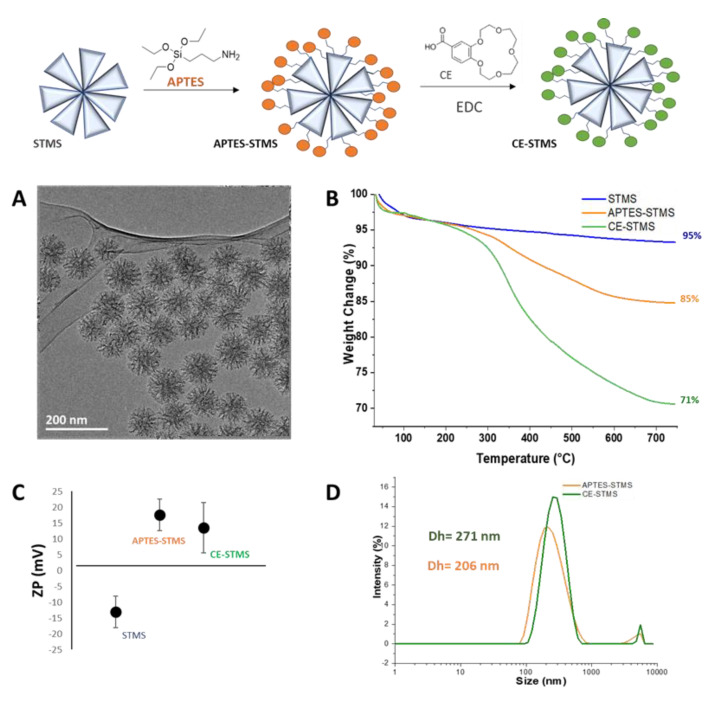
Top scheme: principle of CE grafting on STMS. (**A**) TEM image of STMS NPs, (**B**) TGA and (**C**) ZP of STMS (blue), APTES-STMS (orange), CE-STMS (green), and (**D**) DLS size distributions of APTES-STMS (orange) and CE-STMS (green).

First, STMS NPs (Figure 1A) were obtained according to a sol-gel process consisting of the hydrolysis and condensation of a TEOS precursor in the presence of CTATOs surfactant, as described previously [26,27].

TEM imaging analysis indicated an average size of STMS nanoparticles of 105 ± 7 nm and a large pore size in the range of ca. 10 nm. In previous studies, nitrogen isotherm adsorption–desorption experiments reported a pore size of ca. 11–15 nm and surface areas of ca. 400–600 m^2^·g^−1^ [31,36]. Then, to ensure the grafting of the crown ether (CE) bearing carboxylic groups via peptidic-like coupling, the STMS NPs were functionalized with APTES to exhibit amine-reactive groups. This reaction was carried out in ethanol where the STMS particles were well dispersed in the presence of ammonia (Stöber-like conditions) to form a siloxane-ammonium layer. TGA results (Figure 1B, APTES curve) and ZP values (Figure 1C) led to 129 µg_APTES_·mg_SiO2_^−1^ and +16 mV, respectively, values which are in agreement with previous works [28,31].

For CE grafting, the acidic functions on the chelating ligands were activated by the carbo-diimide activator agent EDC forming a reactive O-acylisourea intermediate product, unstable in an aqueous solution, which reacted with the amine functions on the APTES-STMS surface (Scheme S1, Supplementary Materials). Then, the primary amine grafted on the silica surface formed an amide bond with this previously activated carboxyl function [37]. TGA quantification (Figure 1B, CE green curve) allowed us to determine the grafting amount of this organic molecule: first, solvent weight loss was observed at 100 °C (weight loss ca. 5%); then, between 350 °C and 680 °C, continuous weight loss was detected due to the presence of APTES and CE molecules grafted on the silica surface (weight loss ca. 29%). From these weight losses, the grafting rate was deduced to be about 223 µg_CE_·mg_SiO2_^−1^, corresponding to 742 nmol_CE_·mg_SiO2_^−1^ and 0.9 ligand·nm^−2^. A similar grafting rate was also found by Azaroon et al. [38], who grafted 18-CE-6-Pd on MCM-41; they succeeded using another method to obtain ca. 288 µg [18-CE-6]·mg_SiO2_^−1^ (732 nmol_EC_·mg_SiO2_^−1^). After CE grafting, the ZP value of the functionalized STMS did not show any change (ZP = +15 mV), in agreement with the absence of charge in the CE molecule and of some remaining amine functions. Figure 1D demonstrates that the CE-STMS suspensions display good colloidal stability with an average hydrodynamic size of ca. 271 nm.

#### 2.1.2. Sodium Capture with Crown Ether Grafted STMS Nanoparticles

Then, we addressed the ability of CE-STMS to chelate sodium in two different media: in a methanol–water (80/20% *v*/*v*) mixture to investigate the sodium capture in optimal conditions and in pure water to work in conditions close to dialysis applications. Indeed, studies and experiments performed on free CE (not grafted) showed better constant stability in organic solvents or co-solvents than in water (log K_water_ = 0.58, log K_THF_ > 4 [39], log K_acetonitrile_ = 4.91 [40], log K_acetone_ = 3.68, log K_DMF_ = 1.97 [25]). According to the study of Dishong, who studied the variation of the stability constant of the complex *Na15EC5* in different proportions of a mix of methanol–water, the co-solvent chosen was a mix of 80/20% (*v*/*v*) methanol–water [41].

To quantify the sodium removal by CE, the following parameters were introduced (same equations for the cryptand experiments):

$$\mathrm{Capture}\;\mathrm{Capacity}=\frac{n_{\mathrm{Na}-\mathrm{captured}}\;(\mathrm{nmol})}{m_{\mathrm{silica}}\;(\mathrm{mg})},\;\mathrm{Capture}\;\mathrm{Efficiency}(\%)=100\;\times \;\frac{n_{\mathrm{Na}\;\mathrm{captured}}\;(\mathrm{mol})}{n_{\mathrm{Na}\;\mathrm{initial}}\;(\mathrm{mol})},\;\mathrm{Macrocycle}\;\mathrm{Coverage}\;(\%)=100\;\times \;\frac{n_{\mathrm{Na}-\mathrm{captured}}\;(\mathrm{mol})}{n_{\;\mathrm{grafted}\;\mathrm{macrocycles}}\;(\mathrm{mol})}.$$

We thus tested the sodium capture by the CE-STMS in this co-solvent (80:20) for two molar stoichiometries, Na:CE 0.9:1 and 4.7:1. The results summarized in Table 1 show that sodium capture was improved, from a capacity capture of 245 nmol_Na+_·mg^−1^_SiO2,_ for Na:CE 0.9:1 stochiometry, to 1600 nmol_Na+_·mg^−1^_SiO2_ for Na:CE 4.7:1 stochiometry. These values correspond, respectively, to a CE_grafted_ coverage of 25% and 216%. 

Control experiments with APTES-STMS, i.e., without CE grafted, were achieved in the same conditions for sodium capture (Table S1, Supplementary Materials, for MeOH:water (80:20) case). Results indicated limited sodium capture (56 and 0 nmol_Na+_·mg^−1^_SiO2_). This shows that the grafting of CE at the STMS surface ensures highly specific metal chelation achieved by the macrocycle. Moreover, compared to free CE, grafting does not affect the capacity to efficiently chelate sodium cations. Experiments in a co-solvent environment and large excess of sodium (4.7:1) suggest an occupancy of Na^+^ of 216% of grafted crown ether, much more than the initial vacancy sites. An explanation could lay on additional interactions in this co-solvent between NaCl ion pairs and the modified porous STMS NPs. Hence, the trend observed in Na^+^ capture by CE-STMS can be explained by two mechanisms: one majority corresponds to strong and selective chelation binding with metal–ligand coordination bonds, and the other, non-selective and potentially corresponding to weak interactions with the modified-STMS surface.

With the aim of applying our nano-objects for peritoneal dialysis applications, we also assessed sodium capture in aqueous solutions at different pHs (pH = 7 and 5) and at different Na:CE stoichiometries (0.9:1 and 4.7:1). In water for a 0.9:1 stoichiometry condition at pH = 7, a low capacity capture of 28 nmol_Na+_·mg^−1^_SiO2_ (corresponding to 2.8% of efficiency and to 3.7% of coverage) was observed. In order to displace the sodium capture equilibrium, CE-STMS NPs were introduced in a solution with a larger excess of sodium at a stoichiometry Na:CE 4.7:1 at pH = 7, which yielded the improvement of the capacity capture to 124 nmol_Na+_·mg^−1^_SiO2_ (corresponding to 16.7% of coverage). Similar experiments in an acidic solution (pH 5) did not allow for improving this result as 108 nmol_Na+_·mg^−1^_SiO2_ were obtained (14.5% of coverage). Control experiments with APTES-STMS were also achieved in water in the same conditions for sodium capture (Table S1, Supplementary Materials for H_2_O case). Results indicated limited sodium capture (1, 0, and 11 nmol_Na+_·mg^−1^_SiO2_), which agrees with a pure repulsive interaction between sodium cations and the positively charged STMS surface. This clearly demonstrates the highly specific capture of sodium by the grafted CE macrocycle. Moreover, by comparison with the MeOH:water co-solvent, the lower amounts of captured sodium are strongly correlated to the lower constant stability of CE in water.

Thus, functionalized CE-STMS NPs were shown to be less efficient in capturing a consequent quantity of sodium in water, even with more sodium stoichiometry as compared to organic media. According to studies of ion uptake with crown ethers, to improve sodium capture, solvents other than water are able to form a more stable complex for two main reasons. The first one is that the structure of the complex is upgraded by the addition of solvent molecules to complete the complex structure (log Ks_Na-CE in water_ = 0.58, log Ks_Na-CE in chloroform_ = 3.25) [34]. The second one is that cations are more strongly solvated in water than methanol; consequently, the solvation sphere increases the cation radius, and the cation–cavity match becomes more difficult [42]. This could explain the behavior of our system; nevertheless, it is important to emphasize that grafting the CE on a support did not modify the sodium capture ability of the 15-crown-5 ether.

### 2.2. Cryptand Functionalization on STMS and Sodium Extraction

Previous experiments showed the capacity of CE-STMS to remove sodium in two different media. Nevertheless, for peritoneal dialysis, it is impossible to work with 80% methanol due to its toxicity and health problems [43]. To perform our experiments in water, another ligand with a higher constant complex with sodium was considered: cryptands, and in particular, the cryptand C_221_ (log Ks_water_ = 5.40) [35]. Cryptands are suitable for sodium uptake as they possess a higher stability constant in water and other solvents. This is due to the macrobicyclic cryptate effect, by analogy to the macrocyclic effect [42]. However, due to the chemical nature of the molecule, the grafting of cryptands at the surface of NH_2_-STMS is more challenging than that of CE.

#### 2.2.1. Grafting of Cryptand_221_ on STMS Nanoparticles

To covalently graft C_221_ cryptands on STMS NPs, different strategies with different acid activators (namely EDC, EDC/NHS and HBTU) were carried out. Associated reactional mechanisms are provided in SI: EDC (Scheme S1, Supplementary Materials), EDC-NHS (Scheme S2, Supplementary Materials), and HBTU (Scheme S3, Supplementary Materials). The molecule cryptand C_221_ bearing a carboxylic group was fully synthesized according to a multistep procedure.

**Figure 2:**
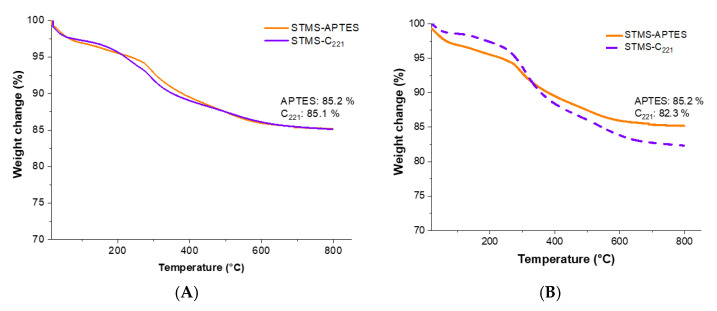
TGA of (**A**) C_221_-STMS (violet) grafted by EDC and (**B**) C_221_-STMS (dash violet curve) grafted by EDC-NHS.

The first C_221_ cryptand grafting method was achieved following the same protocol as with the crown ether chelate (Scheme S1, Supplementary Materials). The reaction with the amines of NH_2_-STMS NPs involved the formation of a peptidic bond to link C_221_ cryptands. As for CE grafting, C_221_ grafting was evaluated by thermogravimetric analysis (Figure 2A, C_221_ violet curve) at 1.2 µg of C_221_ per mg of STMS (2.7 nmol_C221_·mg_SiO2_^−1^), which is very low grafting. It may be possible that the cryptand, being a bigger molecule than CE, has a more steric constraint, and its reactivity is decreased. It is possible that the intermediary product O-acylisourea was not stable enough in time and did not meet an amine group during the reaction.

In order to use a more stable intermediate molecule, the EDC-NHS method was applied. In general, N-hydroxysuccinimide (NHS) is included in the protocol to improve the reaction efficiency and to form a more stable amine-reactive intermediate. In Scheme S2 (Supplementary Materials), the O-acylisourea couples NHS to carboxyls; then, a sulfo-NHS-ester is formed that is more stable (from minutes to hours) than the O-acylisourea. This should allow an efficient conjugation of primary amines at physiologic pH [37]. Figure 2B shows a slight improvement in the grafting yield with a grafted amount of 35 µg of C_221_ per mg of STMS (80 nmol_C221_·mg_SiO2_^−1^). Nevertheless, the result can still be improved.

To improve the grafting, it was assumed that the cryptand cages must be far from the silica, so the addition of longer chains (i.e., other polymers) could help to obtain greater grafting rates. Hence, to increase the number of flexible amine groups at the surface of STMS and increase the flexibility of the chains, we changed the APTES functions by poly(ethylenimine), PEI: a polymer containing primary and secondary amine functions. As described in Scheme S3 in Supplementary Materials, the carboxylic acid of C_221_ was first deprotonated with a strong base (triethylamine). Then, the carboxylate group attacked the imide carbonyl carbon of the HBTU. The activator was HBTU, well reported to lead to a more stable intermediate group. An intermolecular arrangement provided a HOBT^−^ anion and an ester. Later, HOBT^−^ attacked this ester to create the activated leaving group HOBT-ester. Finally, amine groups of PEI-STMS displaced the HOBT^−^ and the C_221_ cryptand was grafted on the surface by a peptide bond.

**Figure 3:**
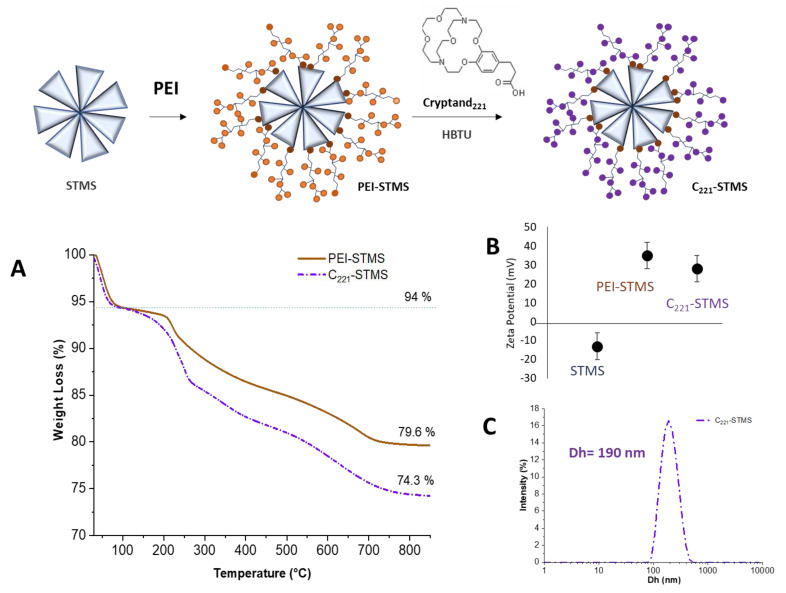
Top scheme: principle of C_221_ cryptand grafting on STMS. (**A**) TGA of PEI-STMS (brown curve) and C_221_-STMS (dotted violet curve) grafted with HBTU. (**B**) Zeta potential of the samples. (**C**) DLS size distribution of C_221_-STMS.

In the first rapid and simple step, the STMS was functionalized with PEI via a simple procedure of electrostatic adsorption. In Figure 3A, TGA analysis indicates that PEI-STMS and C_221_-STMS experienced weight loss at 100 °C, and as for other samples, it was attributed to the evaporation of the solvent (water). The other observed weight losses between 200 °C and 730 °C were attributed to the burning of grafted organic molecules (polymer chain, ammonium groups). The grafting rate was calculated to be at about 181 µg_PEI_·mg_STMS_^−1^. In the same way, TGA analysis (Figure 3A, C_221_ violet curve) allowed us to determine a grafting rate of about 96 µg of C_221_ per mg of STMS (219 nmol·mg^−1^).

The zeta potential (ZP) value evolution was in agreement with the sequential steps of the functionalization (Figure 3B): bare (ZP = −13 mV); PEI (ZP = +35 mV); C_221_ modified STMs (ZP = +28 mV). These values were consistent overall with the incorporation of ammonium groups and non-charged molecules (C_221_). Finally, the C_221_-STMS formed excellent colloidal suspensions in water with a mean hydrodynamic diameter (Dh) of ca. 190 nm (Figure 3C).

#### 2.2.2. Sodium Capture with C_221-_Grafted STMS Nanoparticles in Water Media

Contrary to the study with CE-STMS NPs, experiments with C_221_-STMS (219 nmol_C221_·mg_SiO2_^−1^) were directly performed in water. Sodium capture was performed in aqueous solutions at different Na:CE stoichiometries (0.9:1 and 4.7:1) and at the same pH = 7.

In water for a 0.9:1 stoichiometry condition, the capture capacity was 34.1 nmol_Na+_·mg^−1^_SiO2_ (corresponding to 12.5% of efficiency and to 15.5% of coverage). Compared with the previous results of CE-STMS obtained with the same stoichiometry 0.9:1 (Na:macrocycle_grafted_), the C_221_-STMS was more performant (34.1 nmol_Na+_·mg^−1^_SiO2_ associated with 12.5% efficiency for C_221_ cryptand vs. 28 nmol_Na+_·mg^−1^_SiO2_ associated with 2.8% efficiency for CE). When using a larger excess of sodium (Na:C_221_ 4.7:1), the capture capacity increased up to 368 nmol_Na+_·mg^−1^_SiO2_ (corresponding to 26.2% of efficiency and 168% of cryptand coverage). Control experiments without C_221_ grafted, i.e., with PEI-STMS, were also achieved in water in the same conditions for sodium capture (Table S2, Supplementary Materials). Results indicated a very limited amount of captured sodium (respectively, 1.5 and 0 nmol_Na+_·mg^−1^_SiO2_). As previously observed, these results demonstrate a highly specific capture of sodium by the grafted C_221_ macrocycle but also additional nonspecific sodium cation adsorption at higher stoichiometries by the chemically modified porous STMS NPs. Finally, these last capture results are higher than for CE-STMS under the same conditions. All these results are summarized in Table 2. These nanomaterials would have, therefore, a high potential for sodium uptake in the peritoneal dialysis treatment. In order to validate their future applications, especially regarding their selectivity, the next experiment aimed at testing C_221_-STMS in multielement media.

#### 2.2.3. Sodium Selectivity of the C_221_-STMS Grafted STMS Nanoparticles

The chelation selectivity of Na^+^ with respect to other cations present in the dialysis solution (Ca^2+^ and Mg^2+^) was studied to evaluate if C_221_-STMS NPs can selectively uptake Na^+^ in a multi-element environment. Two different media were used to demonstrate the sodium capture without other ion perturbation. The first medium is a multi-element solution where all cations have the same molar concentration: Na^+^, Ca^2+^, and Mg^2+^ (2.0·10^−4^ M) at pH 7, corresponding to 0.9 molar equivalents as compared to C_221_ grafted on STMS ([C_221-grafted_] = 2.2·10^−4^ M). The second medium mimics a biomedical situation of peritoneal dialysis using the dialysis solution from Fresenius Medical Care that is provided to patients [44]. It contains Ca^2+^ and Mg^2+^ cations at 2.2·10^−6^ and 8.7·10^−6^ M, respectively. To simulate a high level of sodium cations corresponding to renal failure diseases, the Na^+^ concentration was set at 5.5·10^−4^ M. This concentration is justified by the studies of Fischbach et al. [45]. In their research about sodium removal, they adapted automatic peritoneal dialysis treatment and removed around 20% of the initial sodium concentration. The objective was to reproduce a similar removal efficiency using cryptand-grafted stellate mesoporous silica. The results are summarized in Table 3. 

For the multi-element solution at equimolar conditions, the capture capacity, efficiency, and cryptand coverage (5.9 nmol_Na+_·mg^−1^_SiO2_, 2.2% and 3%) of sodium were smaller than in the previous experiments with C_221_ alone. Moreover, it was observed that calcium capture was much higher than for sodium, with a capture capacity of 65.5 nmol_Ca2+_·mg^−1^_SiO2_ (with capture efficiency and cryptand coverage of 24 and 30%, respectively). These differences were explained by a competition between Ca^2+^ and Na^+^ cations because of their complex constants, the one of Ca-C_221_ being slightly higher than those of Na-C_221_. Concerning magnesium cations, the results agree with the smaller constant complex (capture capacity 11.3 nmol_Mg2+_·mg^−1^_SiO2_). Nonetheless, the capture of magnesium was close and even higher to the one of sodium. This last result may be explained by a change in the complexation constants induced by the aromatic ring in the C_221_ cryptand. In fact, the effects of benzo and other substituents on the complexation properties were reported to decrease metal ion binding and selectivity. A performed study in cryptand [2.2.2] (well known for a high binding K^+^) exhibited a decrease of log Ks values (from 10.49 to 9.21) and a loss of selectivity of K^+^ [46]. The previously reported values of complexation constant were of the naked cryptand, but our cryptand also contained a benzene ring in the structure. Therefore, it is possible that the affinity of our cryptand was different. Instead of the expected selectivity, Ca^2+^ > Na^+^ > Mg^2+^, the selectivity was modified and became Ca^2+^ > Mg^2+^ > Na^+^. Another interesting result here was the total cryptand coverage. If the three results were considered, total coverage of 38.1% was reached, with many cryptand cages still being free. Indeed, if there was differing reactivity of the ligands based on the complex constants and if it was easier to capture more calcium than sodium, the uptake might have also been highly dependent on cation concentrations.

Furthermore, it is worth noting that, even if the capture efficiencies for Ca^2+^ and Mg^2+^ are higher than for Na^+^, the cryptand C_221_ remains, nevertheless, the best ligand to capture sodium to the best of our knowledge. Indeed, Ca^2+^ and Mg^2+^ being present in ultra-small amounts in the peritoneal medium or in blood might not interfere with sodium uptake. As sodium is the most concentrated species of this medium as compared to Ca (ratio Na/Ca = 250 times) and Mg (ratio Na/Mg = 63 times), its capture could be improved despite the affinities for Ca and Mg. Thus, regarding the study in the medium of peritoneal dialysis, achieved at 2.6:1 eq. of Na:C_221_, the results showed a higher capture for sodium than the other cations (capture capacity of 20.7 nmol_Na+_·mg_SiO2_^−1^ vs 0 and 0.75 nmol nmol·mg_SiO2_^−1^ for Ca^2+^ and Mg^2+^)^.^ As compared to sodium alone described above, achieved at 0.9 equivalents, it is less than the amount at 34.1 nmol_Na+_·mg_SiO2_^−1^. The decrease in sodium removal efficiency could be an effect of the presence of calcium and magnesium. Even if the sample presents a very low quantity of calcium and magnesium, their presence could disturb the behavior of the cryptands.

Therefore, the C_211_-STMS particles were shown to be efficient in removing sodium from media containing other cations when the amount of sodium is higher than that of other elements. The presence of calcium in the media affected the sodium removal efficiency, but the low concentration of calcium, as in the peritoneal dialysis conditions, showed a good removal efficiency of about 9% of sodium. This corresponds to the predicted half value (20%) with the adapted automatic peritoneal dialysis of Fischbach et al., who used several cycles of solutions dwells [45,47].

## 3. Experimental Section

Products. 2-amino-2-hydroxymethyl-1,3-propanediol (AHMPD, ≥99.9%), cetyltrimethylammonium tosylate (CTATos, ≥98.0%), aminopropyltriethoxysilane (APTES, 99%) and 4-carboxybenzo-15-crown-5 (CE) were purchased from Sigma Aldrich. 1-ethyl-3-(3-dimethylaminopropyl)carbodiimide hydrochloride (EDC, >98%) was purchased from Tokyo Chemical Industry (TCI). Ammonium hydroxide (NH_4_OH) and 1thylenediaminetetraacetic acid (EDTA) were obtained from Fluka. N-Hydroxysuccinimide (NHS, 98%), tetraethyl orthosilicate (TEOS, ≥99.0%), and branched polyethyleneimine (PEI, M.W. 50,000–100,000, 30% w/w aqueous solution) were purchased from Alfa Aesar. N,N,N′,N′-Tetramethyl-O-(1H-benzotriazol-1-yl) uronium hexafluorophosphate (HBTU, >98%) was obtained from Sigma Aldrich, and N,N-Dimethylformamide (DMF, pure) and methanol were purchased from Carlo Erba. NaCl was obtained from Carlo Erba (technical grade), CaCl_2_ (≥97%) was obtained from Prolabo, and MgCl_2_·6H_2_O (reagent grade) was purchased from Merck. 

Synthesis of stellate mesoporous silica nanoparticles (STMS). STMS NPs were obtained according to a procedure described previously [28,31]. To summarize, 0.436 g of AHMPD and 3.80 g of CTATos were dissolved in 200 mL of distilled water in a 500 mL flask at 80 °C up to complete dissolution (about 1 h). Then, 30.2 g (~33 mL) of TEOS were added to the solution, and the mixture was agitated for 2 h at 80 °C. The created white precipitate of stellate mesoporous silica denoted STMS was filtered under vacuum, then it was rinsed three times with distilled water and dried during ca. 16 h (overnight). The resulting dried powder was calcined at 600 °C for 6 h to eliminate the CTATos surfactant. Upon calcination, a mass of around 5 g of this white powder was obtained and crushed with a pestle and mortar. Well-dispersed STMS NPs were then obtained after several cycles of vortexing/sonication in ethanol and crushing again of the falling precipitate in the tube bottom. Then, the supernatant corresponding to well-dispersed STMS was isolated from the precipitate. 

STMS functionalization with aminopropyltriethoxysilane (APTES). In total, 250 mg of STMS in 25 mL ethanol (10 mg·mL^−1^) were poured into a solution of 7 mL of ethanol in a tube mixed with 1.2 mL of NH4OH. After 5 min stirring, 5 mL APTES were poured and agitated for 2 h. Then, the tube was separated into two parts and STMS-APTES of each part was rinsed twice with 15 mL ethanol before re-dispersion in 20 mL of ethanol. 

### 3.1. Crown Ether Experiments: Grafting on STMS and Sodium Capture

#### 3.1.1. Grafting of Crown Ether (CE) 

The previously-grafted amino STMS were reacted with the carboxyl functions of carboxybenzo-CE, prealably activated with EDC through sequential additions in a HEPES buffer (pH 7.2). For that, 100 mg of STMS-APTES were dispersed in 20 mL of HEPES buffer at pH 7.2 (50 mM, buffered with diluted NH_4_OH solution), then 80 mg of CE were added, and the solution was stirred for 5 min. Then, 234 mg of EDC were added to the solution, and this step was repeated every hour (5 consecutive times). After the last addition, the reactional media was left on the stirring wheel for a night (17 h), then the tube was centrifuged (10,000× *g*, 10 min) and washed 3 times with distilled water. A part of the synthesis was dispersed in methanol, and the other in water. 

#### 3.1.2. Sodium Capture with CE-STMS

Co-solvent media. In total, 6 mg of the CE-STMS (4452 nmol of CE_grafted_) nanoparticles were, respectively, dispersed in 4 mL of methanol–water (80/20% *v*/*v*) media. Two NaCl solutions containing 0.9 molar equivalent of Na ([Na] = 1.1·10^−3^ M) or 4.7 molar equivalents of Na ([Na] = 5.5·10^−3^ M) for 1 equivalent of CE were evaluated. Solutions were stirred for 24 h at room temperature (25 °C) and centrifuged at 12 000× *g* for 20 min.

Water media. 6 mg of the functionalized nanoparticles (4452 nmol of CE_grafted_) were added to a centrifuge tube solution containing 6 mL of 0.9 equivalent of Na for 1 equivalent of CE_grafted_ at pH 7 ([Na] = 7.1·10^−4^ M). The same process was repeated for 4.7 equivalent of Na at pH = 5 and at pH = 7 ([Na] = 3.7·10^−3^ M). The whole was stirred continuously overnight and finally centrifuged at 12,000× *g* for 20 min. The supernatants were analyzed by induced coupled plasma atomic emission spectroscopy analysis with a Varian 720 ES instrument (ICP-AES).

### 3.2. Cryptand Experiments: Grafting on STMS and Sodium Capture

#### 3.2.1. Preparation of Cryptand_221_ and Grafting Studies

**Scheme 2:**
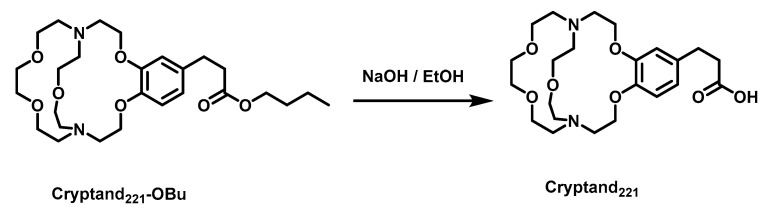
Synthesis of Cryptand_221_ from its butyl ester precursor Cryptand_221_-OBu.

Cryptand_221_ was prepared from the hydrolysis of its tertiobutyl-ester precursor, i.e., Cryptand_221_-OBu prepared from experimental procedures described in the literature (Scheme 2) [48]. Cryptand_221_-OBu was characterized by ^1^H NMR and mass analysis. ^1^H NMR (400 MHz, CDCl_3_) δ 6.82–6.56 (m, 3H), 4.23–3.92 (m, 6H), 3.90–3.82 (m, 1H), 3.81–3.28 (m, 12H), 3.21–2.72 (m, 10H), 2.61–2.37 (m, 5H), 1.59–1.46 (m, 2H), 1.39–1.24 (m, 2H), 0.85 (t, *J* = 7.4 Hz, 3H); HRMS (NSI): *m*/*z* calcd for C_27_H_44_N_2_O_7_Na^+^: 531.30407. [*M* + Na]^+^; found: 531.3039. Saponification of Cryptand_221_-OBu was monitored by HPLC, leading to Cryptand_221_, a white powder used in APTES-STMS and PEI-STMS grafting without further purification.

Grafting of Cryptand_221_ by EDC on APTES-STMS. In the same way, 24 mg of STMS-APTES were dispersed in 10 mL of HEPES buffer at pH 7.2 (150 mM, buffered with NH_4_OH). In total, 30 mg of Cryptand_221_ were added first, and the mix was ultrasonicated for 5 min. After, 56 mg of EDC were added to the solution (5 sequential additions spaced by one hour each) and constantly stirred in a wheel for a night (17 h). The solution was centrifuged (12,000× *g*, 20 min), and functionalized STMS was washed 3 times with distilled water, then dispersed in 5 mL of water.

Grafting of Cryptand_221_ by EDC-NHS on APTES-STMS. Following the same previous principle, the amino-grafted STMS were reacted with the carboxyl function of Cryptand_221_ using EDC and NHS to improve the yield of grafting. In the same way, 24 mg of STMS-APTES were dispersed in 10 mL of HEPES buffer at pH 7.2 (150 mM, buffered with NH_4_OH). In total, 30 mg of Cryptand_221_ were added first, and the mix was ultrasonicated for 5 min. After, 116 mg of EDC and 232 mf of NHS were added to the solution (5 sequential additions spaced by one hour each) and constantly stirred in a wheel for a night (17 h). The solution was centrifuged (12,000× *g*, 20 min), and functionalized STMS was washed 3 times with distilled water, then dispersed in 5 mL of water.

Grafting of Cryptand_221_ by HBTU on PEI-STMS. This time, the amino functions were provided by branched polyethylenimine (PEI). For this, 50 mg of STMS were dispersed in 50 mL of water and 100 mg of PEI were added. The tube was stirred for 2 h, and the STMS-PEI was centrifuged (10,000× *g*, 10 min) and washed several times with water. In total, 24 mg of PEI-STMS nanoparticles were dispersed in 5 mL of DMF. In another flask, 22.7 mg of triethylamine, 30 mg of C_221_, and 85 mg of HBTU were well-dispersed in 5 mL of DMF and added to the flask containing the nanoparticles. The whole was stirred in a wheel for a night (17 h). The solution was centrifuged (12,000× *g*, 20 min), and C_221_-STMS were washed with ethanol and distilled water, then dispersed in 5 mL of water.

#### 3.2.2. Sodium Capture with C_221_-STMS

Water media. The protocol applied for STMS-CE functionalized nanoparticles was adapted to STMS-Cryptand NPs. In total, 6 mg of the functionalized nanoparticles (1314 nmol of C_221_ grafted) were, respectively, added to 6.2 mL of a solution containing 0.9 equivalent of Na ([Na] = 1.97·10^−4^ M) at pH = 7 and to 6.4 mL of a solution containing 4.7 equivalent of Na ([Na] = 9.8·10^−4^ M) at pH 7. All the experiments were carried out for 1 equivalent of grafted C_221_. The whole was stirred overnight and finally centrifuged at 12,000× *g* for 20 min.

#### 3.2.3. Na^+^ Selectivity of the C_221_-STMS

These experiments were conducted under two different conditions. In the first approach, the C_221_-STMS NPs were subjected to a solution containing sodium in combination with a set of metal elements used at the same concentration. In total, 6 mg of C_221_-STMS were dispersed in 6 mL of a multielement solution containing several metal salts at the same concentration ([Na^+^] = [Mg^2+^] = [Ca^2+^] = 2.0·10^−4^ M). These cations are usually present in the peritoneal dialysis solution.

In the second approach, 6 mg of the C_221_-STMS NPs were dispersed in 6 mL of an equivalent of a dialysis solution of a second-generation double chamber “sleep safe” from Fresenius Medical Care. This medium was used to remove sodium during peritoneal dialysis ([Na^+^] = 5.5·10^−4^ M (2.6 eq), [Mg^2+^] = 2.2·10^−6^ M (0.01 eq) and [Ca^2+^] = 8.7·10^−6^ M (0.04 eq). The solution was set at pH = 7 with an NH_4_OH solution. For this experiment, no glucose was added (contrary to the real solution) to avoid possible effects. For these two types of experiments, the reaction mixtures were stirred overnight (17 h) at room temperature (25 °C), centrifuged at 12,000× *g* for 20 min, and the NPs were washed twice with Milli-Q water. The supernatants were analyzed by ICP-AES.

### 3.3. Characterizations Methods

#### 3.3.1. TEM

TEM imaging of the STMS silica NPs was achieved using a JEOL 2100 high-resolution TEM microscope operating at 200 kV. It was used to characterize the size and morphology of the NPs. 

#### 3.3.2. Dynamic Light Scattering (DLS) and Zeta Potential (ZP)

The zetasizer Nano ZS from Malvern was used to characterize the properties of colloidal suspensions: the hydrodynamic diameter and the dispersity in size and intensity mode. Measurements of surface Zeta potential were performed to obtain insights into the surface charge of the particles.

#### 3.3.3. Thermal Gravimetric Analysis (TGA)

TGA was performed on a TA SDT 600 instrument to measure the mass loss of the sample when the temperature changed. Consequently, the amount of organic compound grafted on functionalized inorganic STMS nanoparticles can be quantified.

#### 3.3.4. ICP-AES

The measurement of supernatant concentration was performed on a Varian 720 ES instrument. For the co-solvent supernatants, samples were diluted to have less than 5% of methanol in the whole media. In fact, the device is very sensitive to solvents, which could cause measurement interferences. The functionalized STMS nanoparticles with CE or C_221_ in aqueous suspensions were brought in contact with sodium cations solutions in different media. Sodium uptake was quantified by the dosage of the remaining supernatants by ICP-AES, and the analytical concentration of sodium was measured.

## 4. Conclusions

In this work, the versatile potential of STMS functionalization with macrocycles was applied for specific sodium uptake. Highly specific macrocycles bearing carboxylic moieties such as crown ethers (CE) and cryptands (C_221_) were covalently grafted by complementary carbodiimidation reactions on aminosilane-grafted STMS or polyethyleneimine(PEI)-coated STMS, respectively. Very high grafting of CE on STMS (742 nmol_CE_·mg^−1^_SiO2_) was achieved, while for the C_221_ cryptand, satisfying grafting (219 nmol_C221_·mg^−1^_SiO2_) was obtained, this macrocycle bringing more steric constraint than CE.

Regarding the sodium capture capacities with CE-grafted STMS, sodium capture was more effective in a co-solvent media (methanol–water: 80/20% *v*/*v*, 245 nmol_Na+_·mg^−1^_SiO2_, stoichiometry Na:CE of 0.9:1) as compared to water (28 nmol_Na+_·mg^−1^_SiO2_). Furthermore, C_221_ cryptand-grafted STMS showed similar capture capacities to CE-STMS in water with a stoichiometry Na:C_221_ 0.9:1 (34 nmol_Na+_·mg^−1^_SiO2_); however, the molecular coverage was found to be much better (15.5% vs. 3.7%) due to the lower amount of grafted C_221_. At last, the capture of sodium in the multielement (Na^+^, Ca^2+^, Mg^2+^) media at the same concentration showed a great affinity with calcium rather than sodium (65.5 nmol_Ca2+_·mg^−1^_SiO2_ and 5.9 nmol_Na+_·mg^−1^_SiO2_), which is explained by the complexation constants. However, in the mimicking peritoneal dialysis solution, a medium containing the same ions but with a higher concentration of sodium, the NPs showed a higher capture capacity in sodium (20.7 nmol_Na+_·mg^−1^_SiO2_) compared to the other two elements.

This material shows an interesting potential for use in renal failure treatments since deadly hyponatremia (lack of sodium) treatment should not lead to the massive removal of sodium but is more likely to facilitate low capture, ensuring sodium level regulation. As the massive removal of sodium ions would be detrimental to our organism, sodium uptake should be finely controlled, and a good compromise between effective sodium uptake and sodium level regulation must be achieved. In future works, the combination of sequential low uptakes and recyclability appears as a way to finely control this level.

## Figures and Tables

**Table 1:** Capture capacity and efficiency of CE-STMS in two solutions containing different sodium cations under different conditions. (Na^+^: CE_grafted_ stoichiometries (0.9:1 and 4.7:1), impregnation time of one night at 25 °C, pH = 7 and 5 for water experiments, solvent 80/20% (*v*/*v*) methanol–water on 6 mg of functionalized STMS nanoparticles). Control experiments without grafted CE are given in Table S1, Supplementary Materials.

	MeOH:H_2_O (80:20)		H_2_O		
	Na:CE 0.9:1	Na:CE 4.7:1	Na:CE 0.9:1 pH = 7	Na:CE 4.7:1 pH = 7	Na:CE 4.7:1 pH = 5
Capture efficiency (%)	25	31	2.8	2.4	2.1
Capture capacity (nmol_Na+_·mg^−1^_SiO2_)	245	1600	28	124	108
Coverage (%)	25	216	3.7	16.7	14.5

**Table 2:** Capture capacity and efficiency of C_221_-STMS in two different solutions containing sodium cations under different stoichiometry Na^+^:C_221_ (0.9:1 and 4.7:1). Impregnation time of one night (17 h) at 25 °C, pH = 7 on 6 mg of functionalized STMS nanoparticles grafted at 219 nmol·mg^−1^. Control experiments without grafted C_221_ are given in Table S2, Supplementary Materials.

	Na:C_221_ (0.9:1) pH = 7	Na:C_221_ (4.7:1) pH = 7
Capture Capacity (nmol_Na+_·mg^−1^_SiO2_)	34.1	368
Capture Efficiency (%)	12.5	26.2
Cryptand coverage (%)	15.5	168

**Table 3:** Capture capacity, capture efficiency, and cryptand coverage of C_221_-STMS in two different media solutions containing different metal cations. For the multi-element solution, M: C_221_ stoichiometries are 0.9:1. For the mimicking peritoneal dialysis solution, M: C_221_ stoichiometries are 2.6, 0.04 and 0.01: 1, respectively for Na^+^, Ca^2+^, and Mg^2+^. Conditions are: contact time of one night (17 h) at 25 °C, pH = 7 on 6 mg of functionalized STMS nanoparticles.

	Ion	Na^+^	Ca^2+^	Mg^2+^
Multi-element solution	Initial concentration (M)	2.0·10^−4^		
	Capture Capacity (nmol_ion_·mg^−1^_SiO2_)	5.9	65.5	11.3
	Capture Efficiency (%)	2.2	24.3	4.2
	Cryptand coverage (%)	3	30	5.1
Equivalent to peritoneal dialysis solution	Initial concentration (M)	5.5·10^−4^	2.2·10^−6^	8.7·10^−6^
	Capture Capacity (nmol_ion_·mg^−1^_SiO2_)	20.7	0	0.75
	Capture Efficiency (%)	2.8	0	6.4
	Cryptand coverage (%)	9.5	0	0.03
Ks _Ion-C221_		10^5.40^	10^6.95^	<10^2^

## Data Availability

Available on request.

## Supplementary Materials

The following supporting information can be downloaded at: https://www.mdpi.com/article/10.3390/molecules28124622/s1. Scheme S1. Mechanism of cryptand grafting by EDC, Table S1. Control experiments with APTES-STMS in MeOH: H2O (80:20) and in pure water, Scheme S2. Mechanism of cryptand grafting by EDC-NHS, Scheme S3. Mechanism of cryptand grafting by HBTU, Table S2. Control experiments with PEI-STMS in pure water.
