# Supplementary material for: Grafting of Crown Ether and Cryptand Macrocycles on Large Pore Stellate Mesoporous Silica for Sodium Cation Extraction
Source: Molecules. 2023 Jun 7;28(12):4622. doi: 10.3390/molecules28124622 (PMC10301188; doi:10.3390/molecules28124622)
Supplement: Supplementary file 1 [file molecules-28-04622-s001.zip › molecules-2356637-supplementary.pdf]

Supplementary Materials

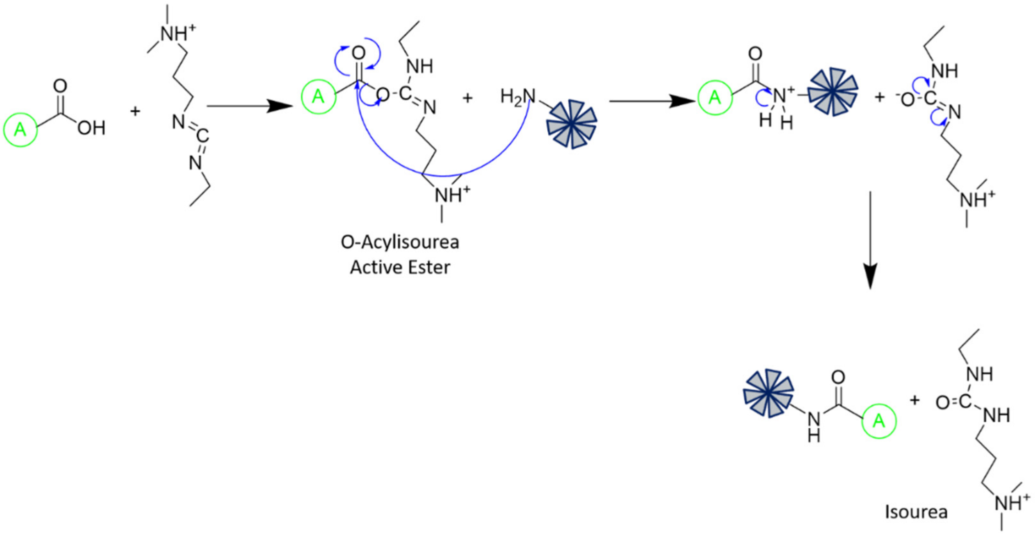

Scheme S1. Mechanism of cryptand grafting by EDC.

Table S1. Control experiments with APTES-STMS in MeOH: H2O (80:20) and in pure water. The value "0" means that the measure is below the detection limit of ICP-AES apparatus (0.07 ppm)

	**MeOH: H_2O (80:20)**		**H_2O**		
	1.1 mM Na^+	5.5 mM Na^+	0.7 mM Na^+ pH=7	3.7 mM Na^+ pH=7	3.7 mM Na^+ pH=5
Capture efficiency (%)	6.2	0	0.13	0	0.6
Capture capacity ($nmol_{Na+}\cdot mg^{-1}_{SiO2}$)	56.7	0	1	0	11

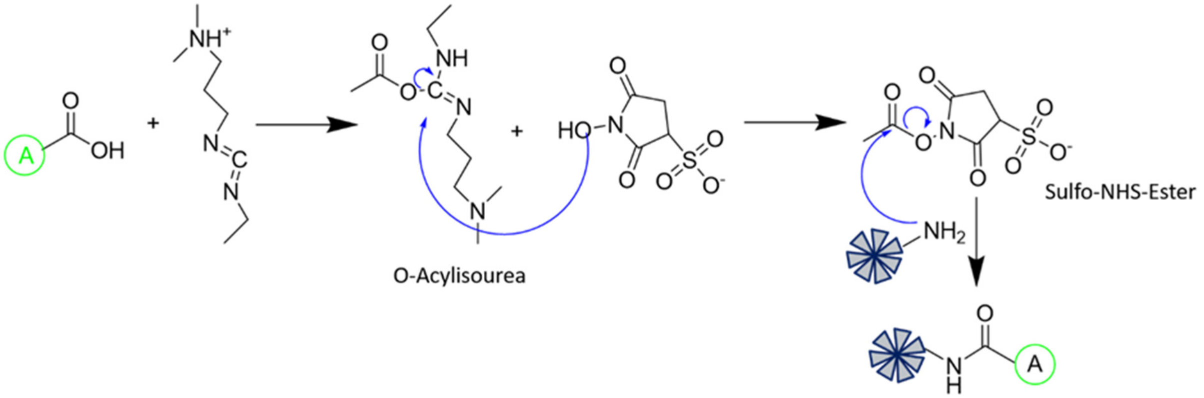

Scheme S2. Mechanism of cryptand grafting by EDC-NHS.

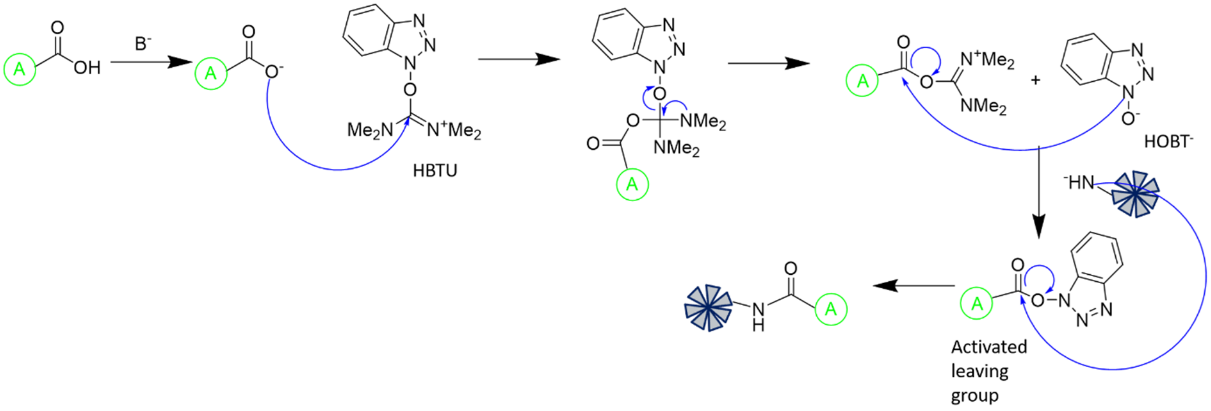

Scheme S3. Mechanism of cryptand grafting by HBTU.

Table S2. Control experiments with PEI-STMS in pure water. The value "0" means that the measure is below the detection limit of ICP-AES apparatus (0.07 ppm)

	0.197 mM Na^+ pH=7	0.98 mM Na^+ pH=7
**Capture Capacity ($nmol_{Na+}\cdot mg^{-1}_{SiO2}$)**	1.5	0
**Capture Efficiency (%)**	0.6	0
